# Processing Ordinality and Quantity: The Case of Developmental Dyscalculia

**DOI:** 10.1371/journal.pone.0024079

**Published:** 2011-09-15

**Authors:** Orly Rubinsten, Dana Sury

**Affiliations:** 1 Edmond J. Safra Brain Research Center for the Study of Learning Disabilities, Department of Learning Disabilities, University of Haifa, Haifa, Israel; 2 Center for the Neurocognitive Basis of Numerical Cognition, Israel Science Foundation, Jerusalem, Israel; Université Pierre et Marie Curie, France

## Abstract

In contrast to quantity processing, up to date, the nature of ordinality has received little attention from researchers despite the fact that both quantity and ordinality are embodied in numerical information. Here we ask if there are two separate core systems that lie at the foundations of numerical cognition: (1) the traditionally and well accepted numerical magnitude system but also (2) core system for representing ordinal information. We report two novel experiments of ordinal processing that explored the relation between ordinal and numerical information processing in typically developing adults and adults with developmental dyscalculia (DD). Participants made “ordered” or “non-ordered” judgments about 3 groups of dots (non-symbolic numerical stimuli; in Experiment 1) and 3 numbers (symbolic task: Experiment 2). In contrast to previous findings and arguments about quantity deficit in DD participants, when quantity and ordinality are dissociated (as in the current tasks), DD participants exhibited a normal ratio effect in the non-symbolic ordinal task. They did not show, however, the ordinality effect. Ordinality effect in DD appeared only when area and density were randomized, but only in the descending direction. In the symbolic task, the ordinality effect was modulated by ratio and direction in both groups. These findings suggest that there might be two separate cognitive representations of ordinal and quantity information and that linguistic knowledge may facilitate estimation of ordinal information.

## Introduction

Using numerical abilities and processing ordinal information can be related to simple daily activities, such as buying a ticket to a movie and finding the correct seat in the movie-theatre. In this example, numbers are used to indicate quantities or magnitudes (e.g. two seats), the identity of something (e.g. seat number five) and the position or rank of an item in a sequence (e.g. the fifth row). The first, quantity processing (of two seats) has been intensively investigated, and findings consistently show that the ability to process quantities is part of a “cognitive core knowledge” associated with evolutionally ancient and specialized cerebral subsystems [1], [2], [3]. The last task, ranking the seats in a sequence, involves the ordinal aspect of numbers [4], [5]. In contrast to the extensive study of quantity processing, to date, the nature of ordinality has received scant attention from researchers, despite the fact that both quantity and ordinality are embodied in numerical information.

In the last 3 decades, it had been extensively argued that the cognitive foundation of mathematics rests on mental representations that developed in the course of evolution [1], [2], [3]. These core representations include a numerical magnitude system that represents the approximate numerical value of a collection of objects [6], representations of space [7] and representations of continuous quantities such as length and time ([8],see also [9]). Here we want to add an additional core cognitive ability: the ability to represent ordered relations. In addition, and based on previous arguments ([10], [11],e.g., [12], [13]), it is hypothesized here that acquired symbolic representations, i.e., language, provides a medium, in which information from these separated core domain-specific systems (i.e., quantity and ordinality) can be combined. Accordingly, new representations that depend on language or acquired symbols are expected to include concepts related to old core or innate components (such as quantity and ordinal information), but which involve also new linguistic combinations (such as the direction of writing).

A major obstacle to the study of cognitive and neural correlates of ordinality lies in the difficulty of teasing apart, at the cognitive level of analysis, processes which are involved-to varying degrees-in both ordinal and quantity processes. Under normal conditions, these processes are inseparably bound. Here we adopted an experimental design [14] that allowed us to differentiate between quantity and ordinal processes (i.e., presenting three numbers as one stimulus, which forces the participants to pay attention to all of the numbers as a triad, as described below). Moreover, to study core ordinal knowledge and since core knowledge of quantities is basically non-symbolic, stimuli in our study were also non-symbolic (group of dots). Specifically, symbolic numerical information involves number words such as “four”, “ten” or “plus”; or written numerical symbols such as “4”, “10” or “+”. On the other hand, non-symbolic numerical information requires non-verbal automatic processing resulting in an implicit understanding of the approximate quantity of concrete sets of objects (e.g., visual dots). Core knowledge of numbers is non-symbolic. Symbolic numerical information, on the other hand, varies across cultures, is influenced by language and arises late in human development (for review see [15]). Hence, the current work studies ordinal processes in both symbolic (Arabic numerals) and non-symbolic (a group of dots) stimuli.

### Ordinality

Can ordinal processing also be considered a core, innate ability? To date, only few studies have dealt with the issue of ordinal processing, in non-human participants and in human infants, and even fewer have investigated the developmental aspect of order processing. Work with infants [16], monkeys [17] young chicks [18] and fMRI in humans [19] [20] suggest that ordinal judging is not exclusively an adult ability, but rather innately available to both humans and animals. This fits with our current hypothesis. Also, these findings indicate that ordinal processing has a biological base and, hence, might also act as a core system.

But is this core ordinal system distinct from the core quantity system? Several recent studies [19], [20], [21], [22] used functional magnetic resonance imaging (fMRI) to compare the neural bases of symbolic ordinality and numerical processing. Collectively, these findings suggest that the IPS, perhaps the anterior region of the IPS in particular, may be involved in the abstract representation of ordinal information that is not number–specific. Hence, there are domain-general representations of ordinal information that are involved in processing any type of stimulus that embodies ordinal information, such as numerical, magnitude and alphabetical stimuli. However, Zorzi and colleagues [23] used support vector machines to reanalyze the data of Fias et al. [21]. They found a clear dissociation between processing numerical vs. alphabetical orders in bilateral horizontal IPS. These findings support previous neuropsychological studies with brain-damaged patients (e.g., [24], [25]), and suggest, in contrast to other arguments,, that ordinal and quantity processing dissociate at both the behavioral and biological levels.

Accordingly, scientific evidence is inconclusive: some evidence suggests that a single numerical magnitude system operates over both quantity and ordinality information, while other sources show signatures of separate cognitive systems for ordinality and quantity processing. One reason for the lack of clarity in previous results (e.g., [22],vs. [25]) could be the use of stimuli used to study ordinal processing. Specifically, in most of the studies in the field of ordinal processing, participants were presented with pairs of items (e.g., numbers, letters, months, etc.) and were asked to decide whether the presentation of these pairs followed an ascending or a descending order [21], [26] or to decide which one of the items appeared earlier/later in a sequence (e.g., [17], [27]). All of these tasks required manipulation of quantity, magnitude or semantic information before extracting order information and arriving at a decision. For example, to know, that 4 and 8 are presented in an ascending and not in a descending order, it has to be initially clear that 8 is larger than 4 (i.e., the context of a numerical comparison must be established). Consequently, it must be assumed that these tasks cannot selectively activate ordinal processing; rather, they require the involvement of several other cognitive processes among which is quantity processing.

We argue here that the question should be whether or not humans are able to implicitly estimate order (as part of core ability) without needing to extract any additional information, such as quantity. Estimation of numbers or quantities relates to the strategy employed when a stimulus configuration is comprised of a large number of items and is presented briefly [28]. It is an intuition available to humans regardless of language and education and, hence, estimation is considered to be part of the core numerical system [6] that is innately available to humans and non-human beings (i.e., animals: [1]). But can we estimate order as well? Do we automatically or unconsciously analyze visual, auditory or any other scene in our daily life based on order as well? Accordingly, to investigate this, we developed a task in which participants are asked to decide if a series of three presented groups of dots or three Arabic numerals are organized in an ordinal fashion or not (without considering whether they are ascending or descending). In the non-symbolic task (i.e., group of dots), no symbolic information is given and counting is not possible due to the brief presentation time.

### Manipulating numerical ratios to indicate numerical representation

One major signature of non-symbolic core numerical representations that is present in human adults, children, infants and non-human animals is that comparisons are subject to a ratio limit: accuracy falls and reaction time (RT) increases as the ratio of the numbers to be compared approaches one ([29], [30],i.e., the ratio effect. e.g., [31], [32]). Similarly, the bigger the distance between two numbers to be compared the faster the response is (i.e., the distance effect. e.g., [15]. For example, Cantlon and Brannon [33] trained monkey and human adults to discriminate stimuli based on their best estimate of numerical value. For both groups accuracy and RT's were modulated by numerical ratio between the stimuli.

Turconi and colleagues [26] compared a numbers comparison task (4–9; which is bigger?) with an ordinal judging task (4–9; ascending or descending order?) and found (1) a reverse distance effect (the smaller the distance is between numbers the faster the response) in the order task and (2) a reduced distance effect in the numbers comparison task when the numbers were presented in an ascending order (4–9). Turconi et al. suggested that the reverse distance effect may reflect specific ordinal related processes, such as serial search or direct recognition of order for sequential numbers. They also suggested that the reduction in the distance effect for ascending pairs in the numbers comparison task may reflect an ordinal related process that involves numbers comparison and may be one of the processes underpinning the distance effect besides magnitude representation.

### Linguistic information that combines the ordinal and quantity systems: Manipulation of ascending vs. descending orders to indicate symbolic order representations

A wide range of work has shown that small-magnitude values are associated with the left side and larger values with the right side of space; (for a recent meta-analysis, see [34]). This effect is known as the spatial numerical association of response codes (SNARC), in which responding to large (compared to small) numbers is faster with the right hand while responding to small (compared to large) numbers is faster with the left hand (e.g., responding to the number “9” with right hand is typically faster than responding to number “1”, regardless of the number's relevance to the task [35]). Such findings have been interpreted as reflecting the influence of directional reading or writing habits. Accordingly, it seems that people also place smaller numbers further on the left side of a mental number line than larger numbers when they enumerate objects or process magnitudes. For example, a smaller SNARC effects was found when participants were Iranians, who habitually read Arabic script from right to left but were only recently immersed into a left-to-right reading culture ([35] experiment 7). Another example is a reverse SNARC effect that was found with Palestinian participants, who read Arabic words and Arabic–Indic numbers from right to left [36].

This may suggest that processing ordered information in general may also be influenced by reading direction and is subject to developmental or cultural and educational influences (i.e., related to the symbolic system) (although, see [37], showing left-to-right preference in nutcrackers and newborn domestic chicks, indicating that at least in part, directional preferences may depend on a biological based system of spatial attention). To note, no SNARC effect was found in children with both arithmetical and visuospatial problems during a number comparison task [38], suggesting abnormal representation of numerical magnitudes on the left-to-right oriented mental number line.

It is argued here that acquired linguistic abilities allow humans to build upon and go beyond our core ordinal or quantity abilities, enabling more advanced numerical concepts such as left-right or right-left orientations. Accordingly, to study symbolic language-based processing of ordinal information, we manipulated the stimuli in our non-symbolic (but also in the symbolic) ordinal task, so that half of the ordered stimuli were presented in an ascending order and the other in a descending order.

Studying ratio vs. direction effect in the ordinality task among individuals who present relatively strong language abilities coupled with a weakness in non-symbolic core numerical processes (e.g., developmental dyscalculia) could indicate not only if ordinal processing is indeed a basic and a separate (from quantity processing) ability, but also how language abilities (e.g., direction) interact with ordinality.

### Developmental Dyscalculia and ordinality

DD is a brain-based disorder, which means that the syndrome-defining cognitive impairment (i.e., deficient calculation skills) is linked to neural deficiencies residing in (intra) parietal brain regions [39]. These deficiencies can be found at the structural [40], [41] and the functional levels alike (DD in children - [42], [43],number line training: [44], [45], [46]); (DD in adults - [47]). The existing brain development imaging literature on DD that focuses on non-symbolic number processing (e.g., comparing the numerosity of two groups of dot patterns) is inconclusive. Studies have demonstrated processing differences between children with and without DD (e.g., [46]) as well as the absence of such differences (e.g., [43]).

Only few studies have investigated performance of DD in comparing non-symbolic numbers. Price and colleagues [46] for example, found differences between DD and controls in symbolic comparisons in an fMRI study. Specifically, the difference was found in brain activation but not in the behavioral results. Also, they found a weak IPS activation in DD children compared to controls when participants compared non-symbolic numerical stimuli. Moreover, Landerl and colleagues [48] found that 8- to 10-year-old DD children were slower than controls in both symbolic and non-symbolic number comparisons. Mussolin, Mejias and Noel [49] found that 10 and 11-year old children with DD show a larger distance effect in both symbolic and non-symbolic numerical comparisons, suggesting deficit in the ability to process numerical magnitudes.

Accordingly, non-symbolic quantity/numerosity processing is suggested by some investigators to be the core deficit of DD [50]. Nonetheless, others, did not find significant differences between DD and controls when processing non-symbolic numerical information [43], [51], [52]. Also, there is an increasing awareness that the core deficit approach, which implies a single-deficit view of DD, is not sufficient to account for the complex and often heterogeneous clinical picture of the disorder [53], [54], [55]. Moreover, and with great relevance to the current work, it could be that the suggested deficit in the innate core system of numerical representation may actually be a core system deficit of order processing. To date, and to the best of our knowledge, only one study investigated ordinal processing in DD, but this study investigated numerical symbolic processes: Kaufmann and colleagues [19] found that in response to symbolic numerical ordinal processing, activation extents in right inferior parietal regions (including the IPS) differed significantly between children with and without dyscalculia. Also in an fMRI numerical training study of DD participants [56] showed how spatial representation of numbers is crucial for the understanding of the principle of ordinality. This finding strengthens the argument that intact development of the IPS is important for the development of ordinal skills.

### The current work

In the current work, we manipulated the ratios between 3 numerical and non-numerical stimuli. We argue that in contrast to previous studies, the brief presentation time and the large number of dots do not allow for serial search or for three separate numerical comparisons. The best way to decide if the three stimuli are ordered or not would be to estimate ordinality (as if using an intuition of order), just like estimating a large number of items. In the current two experiments, we systematically manipulated 5 different variables: (1) DD vs. typically developing participants, to study the possible interaction between deficient core numerical abilities and intact linguistic numerical abilities; (2) Symbolic (Arabic numerals) vs. Non-symbolic (group of dots) representations (in separate experiments), to study linguistic or learnt symbolic effect on ordinality; (3) Ordinality (ordered vs. non-ordered groups of dots or Arabic numerals), to study estimation of order; (4) Direction (ascending vs. descending orders), to study symbolic or culturally influenced orders and (5) Ratio (big or small ratios between the different groups of dots), to study core numerical knowledge. If our hypothesis is correct, we should find not only the typical ratio effect (suggesting numerical processing), but also a main effect of ordinality (i.e., the difference between ordered and non-ordered stimuli) that is independent of ratio. This would suggest a general estimation of order that is independent of the core ability to process quantity information. We also expect to find significant differences between the core numerical abilities (i.e., quantity, as indicated by the ratio effect, and ordinality as indicated by the differences between ordered and non-ordered stimuli) of the DD and control groups. However, if indeed language supports numerical cognition, then direction (left - right vs. right - left orders), which is influenced by linguistic abilities, could modulate the deficient processing of ordinal or quantity information in the DD group. Hence, deficient, ordinal information processing may be enhanced only with the help of linguistic numerical information (e.g., direction).

## Experiment 1

### Non- Symbolic Ordinal Judging: Methods

#### Participants

Twenty-eight native Hebrew speaking adults participated in the study. Fifteen typically developing adults (see Table 1) were recruited through advertisements that were distributed on the Haifa University campus. Also, 13 adults who had been diagnosed with DD (see Table 1) were recruited through a search in the diagnoses database of the clinic for learning disabilities of Haifa University (students diagnosed in the clinic are typically asked to sign a waiver that allows their tests scores to be used for research purposes). In addition, since the use of the database did not produce a sufficient number of participants, advertisements were distributed on the university campus as well as at nearby colleges. All, but one, were right handed.

**Table 1:** Descriptive information and mean percentile scores in the selection tasks for DD and control groups (ACC = Accuracy, RT = Reaction time; m = months, y = years).

	Control group	DD group
*Descriptive information*		
N	15	13
Gender (M/F)	3/12	1/12
Age	25y,9 m (SD = 3y,4 m)	25y, 4 m (SD = 3y,4 m)
*Mathematics*		
Simple calculation-ACC	78	6–17
Simple calculation-RT	60	8
Procedural knowledge-ACC	54–59	2
Procedural knowledge -RT	57	6–10
Numbers line positioning-ACC	46–54	9
Distance relates accuracy	51–70	22–35
Numbers line positioning –RT	55	70
*Reading*		
Text reading-ACC	58–78	58–78
Rapid naming-letters	92–100	67–71
Rapid naming-numbers	70–74	35–44
*Attention (CPT)*		
Omissions	20–38	20–38
Commissions 1	33–67	33–67
Commissions 2	52–81	52–81
RT	52	38–51
Variability of RT	55	39

*Note: Standard deviations are shown in parentheses.*

Participants gave written consent to participate in the experiment and were paid about 30 Shekels for their participation.

#### Ethics Statement

The recruitment, payment, tasks and overall procedure were authorized by the Research Ethics Committee of Haifa University.

#### Classification and assessment criteria

All participants were classified as control or DD, using the “Israeli learning function diagnosis system” (titled in Hebrew also as “MATAL”) for high school and higher education students (National Institute for Testing & Evaluation. For more details, see e.g., [57]). This diagnostic tool is composed of a set of standardized computerized tests and questionnaires intended for diagnosing learning disabilities in high school and higher education students. All tests and questionnaires are nationally normalized.

All participants performed numerical (simple calculation, procedural knowledge calculation and numbers line positioning) tasks, a reading and rapid naming task, and attention (continuous performance test- CPT) tasks. They also answered a questionnaire (based on DSM) regarding their childhood and adulthood attention ability (See Table 1).

The cut-off inclusion threshold was a score below (for the DD group) or above (for the control group) the 20^th^ percentile in either RT or accuracy (ACC) on the simple calculation and the procedural knowledge tests, and a score above the 10^th^ percentile (for both groups) in the reading and attention tests (see Table 1).

#### Experimental task: Non-Symbolic comparisons

The experiment was run on a PC using E-Prime 2.0 software. Participants were presented with three non- symbolic quantities (i.e., 3 groups of dots) in one slide (i.e., one stimuli) and were asked to decide if they were ordered (no matter whether ascending or descending) or not (i.e., no ordinal relation between all three items). The quantities of the 3 groups of dots were ordered in an ascending direction (i.e., small, medium, large), descending direction (i.e., large, medium, small) or in a non-ordered sequence that included two possible presentations: (1) medium, small, large quantities or (2) small, large, medium quantities.

In each stimulus (i.e., 3 groups of dots) we also manipulated the ratio between every two adjacent groups of dots. Accordingly, the ratio was either the same (either a ratio of 0.5 between each pair within the 3 items, or a ratio of 0.6 between each pair) or different. The differing ratios/gaps were presented in a sequence, with decreasing gaps (i.e., a ratio of 0.5 between the first and second items, and a ratio of 0.6 between the second and third items) or increasing gaps (a ratio of 0.6 between the first and second items, and a ratio of 0.5 between the second and third items; see Table 2 for details).

**Table 2:** Numeric values of the groups of dots.

	Fixed ratio									
Ratio	0.6					0.5				
	*First*	*Gap*	*Second*	*Gap*	*Third*	*First*	*Gap*	*Second*	*Gap*	*Third*
	3	*0.6*	5	*0.6*	8	2	*0.5*	4	*0.5*	8
	4	*0.6*	6	*0.6*	9	3	*0.5*	6	*0.5*	12
	5	*0.6*	8	*0.6*	12	4	*0.5*	8	*0.5*	16
	6	*0.6*	9	*0.6*	14	5	*0.5*	10	*0.5*	20

	Changing ratio									
Ratio	Decreasing ratio					Increasing ratio				
	2	*0.5*	4	*0.6*	6	3	*0.6*	5	*0.5*	10
	3	0.5	6	*0.6*	9	4	*0.6*	6	*0.5*	12
	4	0.5	8	*0.6*	12	5	*0.6*	8	*0.5*	16
	5	*0.5*	10	*0.6*	15	6	*0.6*	9	*0.5*	18

#### Stimuli

**Figure 1:**
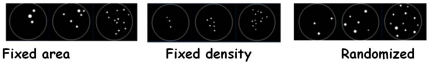
Example of numerical stimuli controlled for area, density and randomized area and density.

Stimuli consisted of 3 groups of multiple-dot patterns ranging from 1 to 20 dots per group. To ensure that participants related to quantities only in the current ordinal task, low-level visual features were excluded. Hence, two experimental blocks of fixed low level visual features were presented in each session: i.e., dot patterns were presented in a fixed area (one experimental block) or with fixed density (in a different experimental block). In a third experimental block in the session, both area and density were randomized (i.e., the random condition; for an example of stimuli presentation, see Figure 1). The three groups of dots in each stimulus were presented along a (non-visible) horizontal axis, with the central pattern located in the center of the screen. For a detailed description of the numerical stimuli and different visual feature conditions, see appendix S1.

#### Procedure

Participants were seated about 60 cm from a 17″ computer screen. Each trial began with a fixation point that flashed for 300 ms. Five hundred ms after the elimination of the fixation point, the sequence appeared and remained in view until the participant pressed a key but no longer than 3,000 ms. The next trial started 1,000 ms after response onset. Participants were asked to decide if the three groups of dots were ordered or not. Responses were indicated by pressing the right hand key for ordinal sequences and the left hand key for non-ordinal sequences. Participants were asked to make their decisions as quickly and as accurately as possible, and were informed that an ordinal sequence could appear in both directions (ascending or descending). A block of 16 practice trials was presented first, followed by nine experimental blocks (three visual feature conditions ×3 repetitions) with a total of 576 trials. Each block contained 64 trials each: 2 directions (ascending, descending,) ×2 orders (ordered and non-ordered stimuli) ×4 ratios (fixed 0.5–0.5, fixed 0.6–0.6, differing 0.5–0.6, differing 0.6–0.5)×4 different stimuli (see Table 2). The three blocks were presented randomly between subjects. The presentation of the sequences in each block of trials was random. The dependent measures were RT and accuracy rates.

#### Data analysis

A series of repeated measures analysis of variance (ANOVA) were carried out on both correct response scores, and the mean RT of correct responses.

### Results

#### Error rate

Mean error rates were low in both control and DD group and hence, detailed analysis was performed only on RT data of correct responses. Specifically, the mean error rate for ordinal sequence in the control group was 4.7% (SD = 1.4, number of trials = 27) and 4.6% (SD = 1.6, number of trials = 26) for the DD group. The mean error rate for non-ordinal sequence in the control group was 6% (SD = 1.5, number of trials = 34) and 7.1% (SD = 1.7, number of trial = 41) for the DD group.

#### RT analysis

Mean RTs of only correct trials were calculated for each participant. We used only trials with RTs that were above 100 ms and below 3,000 ms (accordingly, a total of 62 trials were eliminated from the analysis, 24 trials from the control group and 38 from the DD group). A four-way repeated measures ANOVA was used, which included the group factor (DD or control) and within-group variables of ratio, direction (ascending or descending), ordinality (ordered or non-ordered) and visual features condition (area, density and random). Because Mauchly's Test of Sphericity indicated that the circularity could not be assumed, all of the following *F*-statistics are adjusted by the Greenhouse-Geisser correction.

Results revealed a main effect of the visual features condition [*F* (2, 52) = 25.233, *p*<.0001, 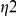 = .493], order [*F* (1, 26) = 8.927, *p*<.01, 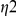 = .256] and ratio [*F* (3, 78) = 98.763, *p*<.0001, 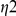 = .792]. Additionally, interactions between order and ratio [*F* (3, 78) = 4.311, *p*<.05, 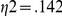]; order and visual features [*F* (2, 52) = 3.175, *p*<.05, 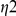 = .109]; visual features and ratio [*F* (6, 78 = 4.803, p<.001, 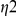 = .156]; and visual features, order and ratio [*F* (6, 156) = 4.019, *p*<.005, 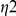 = .134] reached significance.

For the sake of our research questions and hypotheses, it is important to note that the interaction between group and ratio was not significant and it (ratio × group) was even non-significant in any of the visual features' conditions.

**Figure 2:**
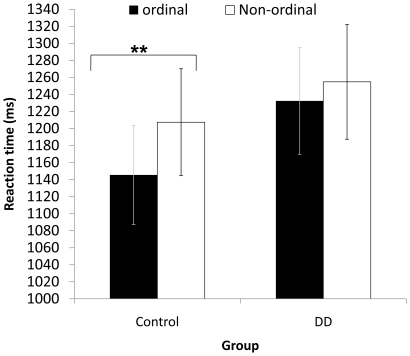
Experiment 1 – non symbolic task. Mean RTs for ordinal and non-ordinal sequences separately for each group. Error bars denote the standard error of the mean. ** p<0.01.

Order and group did not interact, but it is interesting to note that there were different patterns of RTs between the control and DD groups. A separate analysis of each group revealed a main effect for order in the control group [*F* (1, 14) = 11.158, *p*<.005, 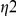 = .444] but not for the DD group (see Figure 2).

Additionally, there was a triple interaction between visual features, order and group [*F* (2, 52) = 3.957, *p*<.05,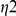 = .132] that resulted from the significant differences between ordinal and non-ordinal sequences in the control group but not on the DD group (see Figure 2).

**Figure 3:**
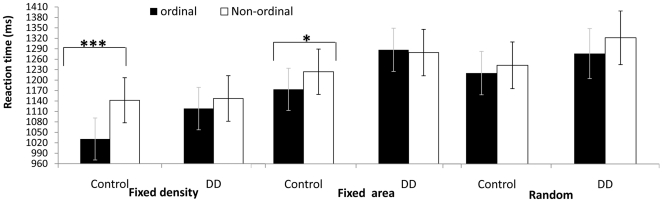
Experiment 1 – non symbolic task. Mean RTs for ordinal and non-ordinal sequences in three visual features condition. Error bars denote the standard error of the mean. * p<0.05; *** p<0.001.

Due to the main effect of the visual features condition and the triple interactions with order and group, we proceeded to analyze each visual feature separately (see Figure 3). The findings were as follows.

#### Fixed density

In the fixed density condition there was a main effect of order [*F* (1, 26) = 13.159, *p*<.001, 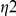 = .336] and ratio [*F* (3, 78) = 34.179, *p*<.0001, 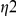 = .568]. Order interacted with group [*F* (1, 26) = 4.526, *p*<.05,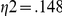]. The source of the interaction between order and group was a significant difference between ordinal and non-ordinal sequences in the control group [*F* (1, 14) = 21.046, *p*<.0001, 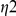 = .601], but not in the DD group (see Figure 3).

*Fixed area:* in the fixed area condition there was a main effect of ratio [*F* (3, 78) = 35.798, *p*<.0001, 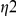 = .812]. The interactions between order and group was marginally significant [*F* (3, 26) = 3.661, *p* = .067, 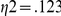], showing a main effect of order in the control group [*F* (1, 14) = 4.601, *p*<.05, 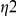 = .247] but not in the DD group.

#### Random condition

In the random condition there was a main effect of ratio [*F* (3,78) = 45.523, *p*<.0001, 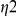 = .636], and interactions between direction and group [*F* (1, 26) = 5.431, *p*<.05, 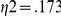]; order and ratio [*F* (3, 78) = 3.295, *p*<.05, 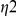 = .112]; direction and ratio [*F* (3,78) = 8.250, *p*<.0001, 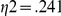]; and order, direction and ratio [*F* (3,78) = 5.551 *p*<.005, 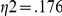].

In a separate analysis of ordinal sequences for each direction (descending/ascending) in the random condition, the descending direction produced a significant effect for order [*F* (1, 26) = 5.997, *p*<.05, 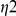 = .208], ratio [*F* (3, 78) = 3.581, *p*<.0001, 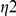 = .606] and an interaction between ratio and order [*F* (3, 78) = 4.860, *p*<.01, 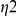 = .139] and ratio and group [*F* (3, 78) = 3581, *p*<.01, 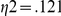].

**Figure 4:**
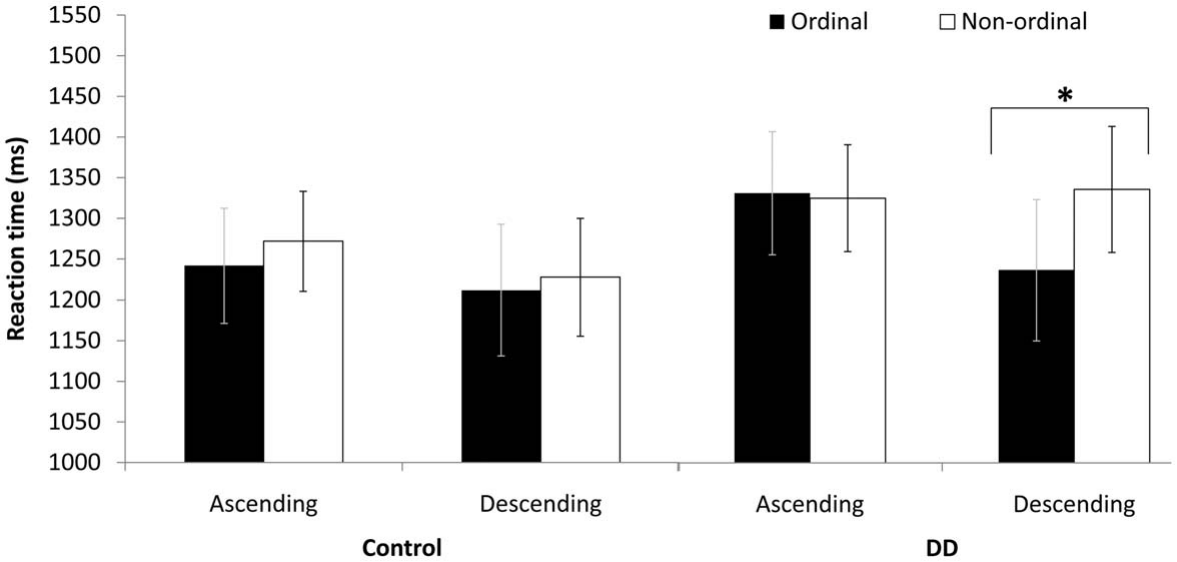
Experiment 1 – non symbolic task, random condition only. Mean RT separately for each group for ordinal and non-ordinal sequences in the different directions. Error bars denote the standard error of the mean. * p<0.05.

Although order, direction and group did not interact, to satisfy a theoretical interest, we continued to analyze the effect of order *separately for each group*. In the DD group, only descending order produced an effect [*F* (1, 12) = 5.225, *p*<.05, 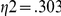 ]; neither direction produced an effect in the control group. In the control group, a triple interaction was noted between order, direction and ratio [*F* (3, 42) = 6.414, *p*<.005, 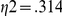; see Figure 4).

### Discussion

The current findings show that DD participants exhibited a normal ratio effect regardless of the presented condition (i.e., constant area, constant density or randomized presentations in the non-symbolic task), suggesting a possible intact “number sense.” In addition, and more importantly, in light of the current working hypothesis, in all cases in which low visual features (i.e., area and density) might have interfered with estimating ordinality, DD participants did not show the ordinality effect. Specifically, to detect an ordinal relationship in the quantitative dimension when total area or density were kept constant, participants needed to ignore systematic changes in the irrelevant low visual features. It has been shown that DD participants may have a general deficit in their ability to ignore irrelevant information, as indicated in several numerical Stroop tasks [58], [59], [60]. However, when low visual features did not infringe on quantitative information and did not act as an irrelevant salient feature (i.e., as in the randomized experimental block), DD participants showed the ordinality effect (significant difference between ordered and non-ordered stimuli) but in the descending (from left to right; which means that the sequence ascends from right to left) direction only. To note, participants were native Hebrew speakers. In the Hebrew language, words are written from right to left and numbers from left to right. Indeed, Shaki and colleagues [36] found a reverse SNARC effect among Palestinians, who read Arabic words and Arabic–Indic numbers from right to left. Accordingly, it could be that written linguistic skills influenced directional preferences in our ordinal task; namely, ordinality effect was found only in relation to stimuli that ascend from right to left (as in the Hebrew language writing system). It is possible that other participants, for example, English speakers, would demonstrate the opposite pattern. This should be tested in future studies. Accordingly, the current findings suggest that if ordinality can be exclusively impaired, separate from quantity impairment, it may function as a separate core ability (in addition to the core quantity module). This core ordinality ability might not have developed efficiently in individuals with DD. Also, acquired linguistic abilities may act as a bridge, such that the use of language combines the core ordinal and core quantitative knowledge.

To further test our hypothesis about the linguistic influence on ordinal decisions, in Experiment 2 we used symbolic (Arabic numbers) stimuli. This enabled us to investigate not only the influence of writing direction on ordinality, but also the influence of actual symbolic knowledge as well.

## Experiment 2

### Symbolic ordinal judging: Methods

#### Participants

Twenty three adults participated in the study. 14 typically developed adults (see Table 3) and nine dyscalculic adults who had participated in experiment 1.

**Table 3:** Mean age and test percentile scores of control participants.

	Control group	DD group
***Descriptive information***		
N	14	9
Gender (M/F)	3/11	1/9
Age	25y,7 m (SD = 3y,4 m)	25y, 08 m (SD = 3y,4 m)
***Mathematics***		
Simple calculation-ACC	78	8–14
Simple calculation-RT	60	3–10
Procedural knowledge-ACC	54–59	5
Procedural knowledge -RT	57	7–9
Numbers line positioning-ACC	46–54	9–11
Distance relates accuracy	51	50–60
Numbers line positioning –RT	55	20–22
*Reading*		
Text reading-ACC	58–78	58–78
Rapid naming-letters	92–100	67–71
Rapid naming-numbers	70–74	33–45
*Attention (CPT)*		
Omissions	20–38	38–100
Commissions 1	33–67	17–33
Commissions 2	52–81	52–81
RT	38	52
Variability of RT	39	55

Higher scores represent better performance. (ACC = Accuracy, RT = Reaction time; m = months, y = years).

Participants gave written consent to participate in the experiment and were paid 30 shekels for their participation. The recruitment, payment, tasks and overall procedure were authorized by the Research Ethics Committee of Haifa University.

All participants were classified as control or DD using the “Israeli learning function diagnosis system” for high school and higher education students, as in experiment 1 (see Table 3).

#### Experimental task

Experimental task and procedure were the same as in experiment 1, other than the stimuli. Participants were presented with a sequence of three Arabic numerals that corresponded with the quantities that had been presented in experiment 1 (see Table 2).

A block of 16 practice trials was presented first, followed by nine additional experiment blocks of 64 trials each: 2 directions (ascending, descending) ×2 orders (ordinal, non-ordinal) ×4 ratios (fixed 0.5–0.5, fixed 0.6–0.6, differing 0.5–0.6, differing 0.6–0.5)×4 sequences. The sequences within the block appeared in a random order. The dependent measures were RT and accuracy rates^1^.

#### Stimuli

Stimuli consisted of 1-digit or 2-digits numbers ranging from one to 20 (see Table 2). The stimuli were white Arabic numbers on a black background, with dimensions of 3 cm length ×3.5 cm width and, presented with a visible circle of 4° visual angle which were created using Photoshop CS4 software.

### Results and Discussion

#### Error rate

Mean error rates were low in both control and DD group; hence, detailed analysis was performed only on RT data of correct responses. Specifically, the mean error rate for ordinal sequence in the control group was 3.7 (SD = 0.9, number of trials = 21) and 4.8 (SD = 1.1, number of trials = 27) for the DD group. The mean error rate for non-ordinal sequence in the control group was 3.6 (SD = 1, number of trials = 20) and 4.8 (SD = 1.2, number of trials = 27) for the DD group.

#### RT analysis

Mean RTs of only correct trials were calculated for each participant, using only those trials whose RTs were above 150 ms and below 2850 ms (a total of 16 trials were eliminated from the analysis, six from the control group and ten from the DD group). A three-way repeated measures ANOVA was used, which included the group factor (DD or control) and within-group variables of ratio, direction (ascending or descending) and ordinality (ordered or non-ordered). As in Experiment 1, all of the following *F*-statistics were adjusted by the Greenhouse-Geisser correction.

Results revealed a main effect of direction [F (1, 19) = 19.441, p<.0001, 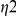 = .506] and ratio [*F* (3, 57) = 44.991, *p*<.0001, 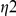 = .703]. To note, there was no significant interaction between ratio and group, suggesting similar ratio effect in both groups. There was an interaction between order and ratio [*F* (3, 57) = 3.393, *p*<.05, 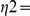.188] and direction and ratio [*F* (3,57) = 8.632, *p*<.001, 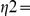.312].

**Figure 5:**
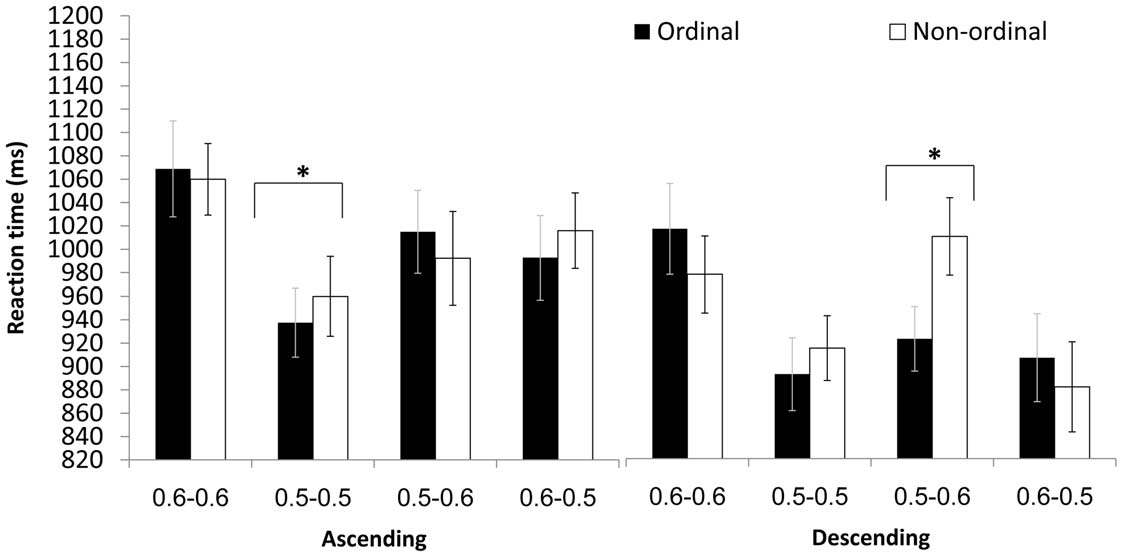
Experiment 2 - symbolic task: Mean RT (for both groups) for ordinal and non-ordinal sequences, in the ascending and the descending direction. Error bars denote the standard error of the mean. * p<0.05.

Analysis of order in each direction separately for each group revealed a significant effect for descending order for the DD group [F (1, 8) = 7.138, p<.05, 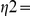.472] and the control group [F (1, 11) = 6.333, p<.05, 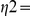.365] but not the ascending order. More specifically, the triple interaction between order, direction and ratio [*F* (3,57) = 7.154, *p*<.005, 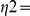.274] was significant. For theoretical reasons we tested this triple interaction (order × direction × ratio) in each group separately: This interaction was found significant for each group separately (for the DD group [F (3,24) = 6.854, p<.0, 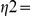.446] and for the control group [F (3, 39) = 3.506, p = .056, 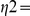.212]; see Figure 5). This suggests that not only direction, but also ratio modulates the ordinal effect.

**Figure 6:**
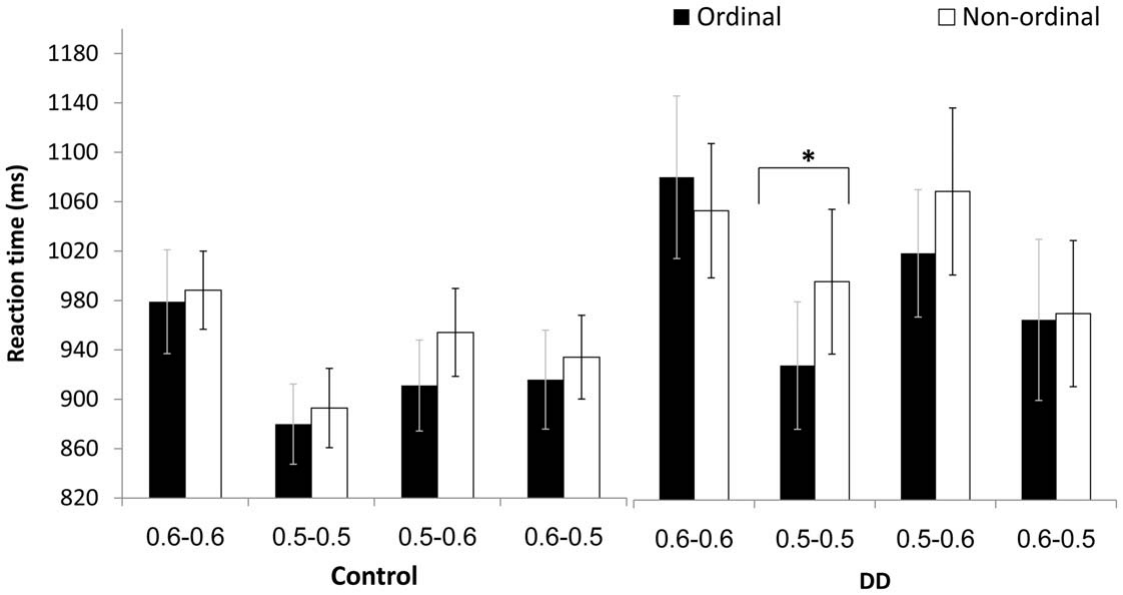
Experiment 2 – symbolic task: Mean RT for ordinal and non-ordinal sequences in four ratios; separately in the control and the DD group. Error bars denote the standard error of the mean. * p<0.05.

In addition there was a triple interaction between order, ratio and group [*F* (3,57) = 3.259, *p*<.05, 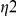 = .146; see Figure 6]. The combination of this triple interaction (i.e., order × ratio × group) together with the triple interaction of order, direction and ratio, which was found to be significant in each group, indicates that both DD and control groups use other clues, such as direction and ratio, to retrieve ordinal information. That is, in the symbolic task, both ratio and direction modulated the ordinality effect in both groups. In addition, ratio modulated general performance similarly in both groups. Interestingly, DD participants used only the larger discrepancy between dot groups (only on trials with the same ratios of 0.5–0.5) to facilitate their ordinal decision, so that the ordinality effect (i.e., a significant difference between ordered and non-ordered stimuli) was noted only in this ratio.

We now turn to the General Discussion to discuss results from both tasks together.

#### Responding with the left hand to ordered sequences

Our results could be influenced by the fact that responding to ordered sequences was done with right hand while responding to non-ordered sequences with the left hand. Accordingly, for each experiment (Exp. 1 and 2 separately) we ran a second block in which the same participants responded to ordered sequences with their left hand and not with their right hand [Experiment 1: 12 of our DD participant (one out of the original 13 did not want to come back) and 13 controls (3 out of the original 15 did not want to come back), Experiment 2: 5 of our DD participants (4 out of the original 9 did not want to come back) and 7 controls]. We did not run a between-subject experiment (in which half of the group responds with right hand to “ordered sequences” and half with left hand) due to a limited number of participants that can be obtained mainly in the pure dyscalculic group.

When we included the “responding hand” as an additional within variable, we found no significant difference between responding with the right hand to ordered sequences vs. responding with left hand in both Experiment 1 [*F* (1, 22) = .009, *p* = .926, 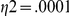] and Experiment 2 [*F* (1, 10) = .761, *p* = .403, 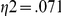]. Also, the responding hand did not significantly interact with the main important interactions that are of interest to our research questions. For example, Experiment 1: no interaction between responding hand, visual features and order [ F (2, 50) = .051, p = .823, 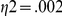], or between responding hand , ratio and order [F (3, 75) = 1.569, p = .204, 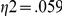. Experiment 2, responding hand did not interact with order and ratio [F (3, 30) = 1.020, p = .398, 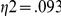] or with direction and ratio [F (3, 30) = .386, p = .764, 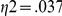]. Moreover, when we analyzed only responding with left hand to ordered sequences, the pattern of results was similar, suggesting that it is not the responding hand that was the cause of our original pattern of results. For example, in Experiment 1: a significant interaction between order and ratio [F ((3, 75) = 3.305, p<.05, 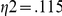] between visual features and ratio [F (6, 150) = 5.6501, p<.0001, 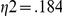] and between visual features, order and ratio [F (6, 105) = 4.764, p<.001, 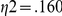]. In Experiment 2 there was marginal significant for direction [ F (1, 10) = 4.034, p = .072, 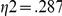 ] and a significant effect of ratio [ F (3, 30) ] = 8.880, p<.005, 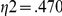].

### Discussion

It was found that DD participants exhibited a normal ratio effect (which is considered to be a signature of magnitude or quantity processes) in the non-symbolic ordinal task, regardless of the perceptual condition (i.e., constant area, constant density or randomized presentations in the non-symbolic task). In the symbolic task, ratio did modulate ordinality more in the DD group than in the control group, suggesting that DD used ratio as a clue to complete the task. In fact, the DD group showed an ordinality effect (i.e., significant difference between ordered and non-ordered sequences) only when the ratio was large and the same (i.e., 0.5–0.5).

A second important finding was that when the DD group performed the non-symbolic task, constant area or constant density of the dots did not modulate the ordinality effect (i.e., the difference in RTs between ordered vs. non –ordered groups of dots). Specifically, our results indicate that DD participants do not show the quantity-based ordinality effect when density and area of the dots are kept constant. However, when both cues (i.e., area and density) are randomized, DD participants, just like the control group, can successfully identify ordinal relations, but only in the descending direction. In contrast, typically developing participants succeed at detecting ordinal relationships of numerical information also when area or density are constant.

A third finding was that, contrary to expectation, in the task that requires acquired linguistic knowledge, as in the case of symbolic stimuli, and which typically renders an exact and not an estimated answer, the ordinality effect was modulated by both ratio and direction in both groups.

Last, it is important to note that the same kind of stimuli were used for manipulating low visual features (i.e., three types of stimuli, which included constant area, constant density, and randomized density and area stimuli) and accuracy was similar between the 2 groups of participants. Accordingly, differences in stimuli complexity could not have contributed to the pattern of results. Therefore, and although DD's eventually do show a low ordinality effect (RT differences between ordered and non-ordered stimuli), the high accuracy rates of the DD group are probably due to other cues that they use, such as linguistic cues (i.e., the direction of “reading” the dots or the Arabic numerals).

We will now discuss (1) the idea of two separate cognitive representations of ordinal and quantity information and (2) the interaction between acquired linguistic abilities (i.e., direction of writing and symbolic representation) and estimation of ordinal information.

### Ordinality and Quantity

One potential explanation for the above pattern of findings may be that there are two separate representations of ordinal and quantity information. When considering this suggestion along with the results showing that DD participants exhibited a normal ratio effect in both the symbolic and non-symbolic tasks, our findings may contribute a novel argument to the literature, namely, that the specific impairment in Dyscalculia may be a deficit in ordinality and not necessarily in quantity processing. Such an argument contrasts with a major alternative view, which assumes that DD results from a core deficit of quantity processing [50]. In particular, in our DD group, the noticeable contrast between the significant effect of ratio and the low and mostly insignificant difference found between RTs to ordered and non-ordered stimuli provides support for invoking two systems.

This notion, of two systems, could be also supported by findings related to the symbolic task. Namely, Arabic numbers are automatically associated with their represented quantities and are learnt in a specific direction (e.g., left to right). Accordingly, the ratios between numbers and their direction (left to right) are two important aspects that influence numerical symbolic representations. When participants are asked to **estimate** ordinality, a task (estimation) that is not natural (for either DDs or control) in the context of symbolic representation, participants use ratios and directions as natural clues to facilitate their ordinal estimations. Again, this may suggest that ordinality and quantity are being processed separately.

Although the current data does not directly support the following claim, it is—nonetheless- tempting to consider the possibility that these two core systems together, quantity and ordinality, when both are intact (as in the control group) may form the foundation for humans' basic “number sense.”

To note, there are several other behavioral experiments with humans and non-human animals, which as in the current work, show signs of separate cognitive systems of ordinality and quantity processing and, hence, support the current idea. For example, it has been shown that young chicks use ordinality and not distance when required to identify a target by its numerical serial position [18]. Also, Zorzi and colleagues [23] found dissociation between processing numerical vs. alphabetical orders in bilateral horizontal IPS, indicating that ordinal and quantity processing dissociate. Moreover, Delazer and Butterworth [24] described the cognitive abilities of SE, an acalculic patient with impaired cardinal numbers but spared ordinal numbers. Specifically, SE, who suffered from a left frontal infarct, was unable to access the cardinal meaning of numbers (i.e., deficiencies in calculation tasks and an inverse distance effect in number comparison), yet was able to answer correctly “which number comes next?” questions, suggesting that the sequential meaning of numbers was preserved. The reverse dissociation was reported by Turconi and Seron [25]. They described a patient with right parietal lesion who was impaired in processing the order of words that denote ordinal information (i.e., numbers, letters, days and months) in various tasks, while showing better performance in processing quantity information. Together, these studies suggest that there are distinct brain and cognitive and maybe also biological structures responsible for quantity and order processing.

Indeed, the current results provide support for the two-system view of numerical cognition (ordinality and quantity) in adults, yet it does not reveal whether the ordinal processing system reflects a core cognitive capacity. Although it is less commonly discussed in the scientific literature, other research studies have shown quite clearly that processing ordinal information may be considered a core cognitive ability (just like the core quantity system) (e.g., [17], [27], [37], [61], [62]).

### Language: A Possible Bridge between Ordinality and Quantity

Our results also suggest that DD participants can retrieve ordinal information just as the controls can, mainly when directional cues are present. Specifically, in the DD group, when area and density were randomized, the ordinality effect appeared only in the descending direction. To note, this fits with the Hebrew writing system, in which words and sentences, are written from right to left. It may be argued that these data support the thesis put forth by Spelke and others ([10], [11],e.g., [12], [13]), namely, that human cognition begins with a set of core systems of knowledge, which for the most part remain constant (either intact or deficient) throughout development. Nevertheless, new representations may emerge when children learn language, because language provides a bridge between these distinct systems and hence, combines the information. The core systems (in this case ordinality and quantity) are limited in terms of the amount and type of information that they can process, that is, they take in some but not all of the sensory information (e.g., [62]). Our results may support such a hypothesis, from the point of view of a deficient core ordinal ability. Specifically, DD participants may have a slight deficit in core ordinal ability, which from early development up to adulthood, does not enable them to use any type of information (such as area) to efficiently retrieve ordinal cues from the world around them. However, with development and acquisition of linguistic skills (e.g., right-left writing system) ordinal information processing is facilitated.

It should be noted that there might be another possible reason why DD participants' responses were not facilitated by the physical and very salient cues of area and density. A critical feature of the task and the design of stimuli in our experiments is that to detect an ordinal relationship in the quantitative dimension, when total area or density are kept constant, participants need to ignore non-monotonic changes in the irrelevant dimensions. For example, the number of dots could increase or decrease, whereas cumulative surface area was held constant and thus dots' size was inversely related to number. The difficulty for DD participants may be in extracting ordinal relations from a single dimension when faced with conflicting changes in other quantitative dimensions. This may not be surprising, given DD typical results in the numerical Stroop task. The numerical Stroop task required the participant to ignore the numerical value of the symbol and decide which number looked physically bigger (e.g., incongruent stimulus: 4 8, the correct response is “4”; congruent stimulus: 4 8, the correct response is “8”; numerically, in both examples the number 8 is larger). Typically, participants are unable to ignore the irrelevant dimension, which interferes with the processing of the relevant one (this is the Size Congruity Effect: The difference between the RTs in the congruent and incongruent conditions). Such a result is typically considered both a failure of the selective attention system and an indication for the automatic nature of the irrelevant dimension (i.e., the numerical value). Rubinsten and Henik [58] found that compared to controls, the DD group showed no size congruity effect in the grayness task (i.e., which of the two numbers is darker, ignore the numerical value), and a significantly smaller effect in the height and physical size tasks. Similarly, Landerl and Kölle [59] found a systematic congruity effect in a physical comparison task for control but not for DD children, (see also [60]).

Unfortunately, our results do not differentiate between these two explanations (i.e., deficient control or attentional system vs. inability to use continuous cues such as area and density). However, our results demonstrate that when area and density information are randomized and cannot facilitate decision, DD participants are better able to compare and contrast three groups of dots based on their ordinal relationships but only with the use of linguistic cues (i.e., direction).

### Conclusions

Collectively, these findings may contradict hypothesis which predicts that DD participants should be impaired in tasks requiring the processing of number magnitude or quantities. Since the DD group's performance was modulated by the ratios between the groups of dots but not by the ordinal aspect of the stimuli (ordered vs. non-ordered stimuli), the overall pattern is in accordance with our view that ordinal deficit–and not necessarily quantity deficit—is characteristic of DD. Further support for this claim may also lead to the conclusion that actually two separate core systems (together with others) form the foundation of numerical cognition: (1) the traditionally accepted numerical magnitude system [6], and also (2) the ordinal system.

The current findings suggest also that typically developing adults can identify ordinal relations that are based on number, with or without the contribution (or interference) of low visual features (i.e., area and density). In contrast, DD participants are unable to use any of these low visual features to estimate order, but instead rely on directional cues (i.e., left to right vs. right to left), which are culturally or linguistically based.

**Figure 7:**
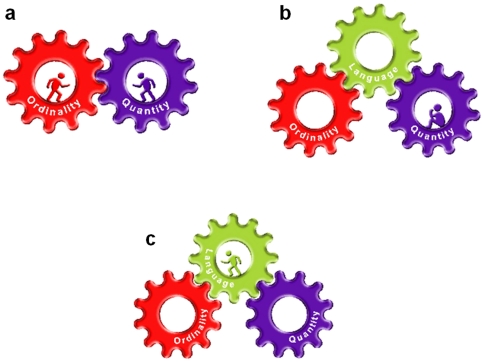
A schematic model of the two core system hypothesis. 7 a. Two separate core systems, ordinality and quantity, that, if intact as in the case of the control group, can work together. 7 b. However, this cogwheel system cannot operate as a single coherent system if one of them is deficient (as in the case of DD in which the ordinal system is deficient; in the model - indicated by the sitting person in the cogwheel). The two separate core systems need a third one, such as language (represented here by direction of writing), to bridge between them in order to operate as a single system. However, the cogwheel that is starting the movement is deficient (indicated by the sitting person) the system will not move and will not work. 7 c. If the cogwheel that is starting the movement is working (e.g., participants are using intact linguistic cues such as direction of writing) as indicated by the running person, this will move the whole system, even if there is one slightly deficient cogwheel.

Visually, these two core systems can be described as two cogwheels (one representing ordinality and the second, quantity) that, when deficient, need a third one (e.g., language) to combine them in order to operate as a system (see Figure 7 a). If one of these cogwheels does not work efficiently, as in the case of DD in which ordinality is deficient, the whole system does not work (see Figure 7 b). However, if individuals with DD use language to operate the system, even the deficient “ordinality cogwheel” moves and the system (i.e., the three cogwheels together) work efficiently (Figure 7 c).

## Supporting Information

Appendix S1 **Detailed description of non-symbolic stimuli.**
(DOC)
