# Supplementary material for: Processing Ordinality and Quantity: The Case of Developmental Dyscalculia
Source: PLoS One. 2011 Sep 15;6(9):e24079. doi: 10.1371/journal.pone.0024079 (PMC3174157; doi:10.1371/journal.pone.0024079)
Supplement: Appendix S1 — Detailed description of non-symbolic stimuli. (DOC) [file pone.0024079.s001.doc]

# **Appendix S1.** Detailed description of non-symbolic stimuli

Stimuli consisted of multiple-dot patterns ranging from 1 to 20 dots per stimuli. Stimuli were generated using a custom-written software programmed in the C# (pronounced as c sharp) language above Microsoft .NET 2 Framewotk™ using Visual Studio 2005 IDE. This software provided the control of parameters of the dot patterns. We used resolution of 1280x1024 to create the images. Dot location in each stimuli were randomized and three sets of stimuli were created; one for controlling area, the other for controlling density and the third set consisted of random density and area of dots (see figure 1 for illustration).

White dots appeared on a black background and were positioned within the bounds of a white circle of a 7° visual angel (VA) (was calculated using formula:
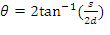
 where *d* is the distance between the subject's eye and the screen and *s* is the size of the object on the screen). Each dot size was randomly varied between 0.17- 1.5 VA. Each dot position was determined by placing it on a randomized arc of an inner circle with a randomized radius using the following formula:
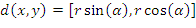
 where
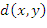
 is the dot position on x,y space, r is a randomized radius from the center of the invisible circle and α is a randomized arc (0-360º) on a circle created with this radius. Randomization method relies on the System's Random library which provides a pseudo random numbers generation method. Each dot was smoothedusing the advanced anti-aliasing algorithm provided with the Microsoft.Graphics2D code library.

The dots never touched each other and were no closer than 0.1 visual angels. This was achieved by randomly selecting a dot location (see randomizing dot location) and comparing the distance of this dot to all the others. If the dot was not closer to another dot than a fixed minimum (1 degree visual angle), the dot was painted. Otherwise, a new position was randomly selected. A maximum number of iterations (5000) were determined as a stop criterion. When the criterion met, the stimuli were omitted and the program wrote an error message for not being able to create suitable dot array for this numerocity.

*Control of Low-level Visual features:* To ensure that the numerosity-discrimination task was solved by judging the discrete quantity, low-level visual features were excluded using control stimuli in addition to standard stimuli. Two sets of control stimuli were used in each session: area and density control

1. *Area matching:* While creating the numeral array, the software calculated the total amount of pixels occupied by the numerals. All stimuli/slides were drawn with the same range of 100 pixel surface. The procedure of matching the stimuli surface was interactional in nature. The software draws an initial array and reduced its pixel size or added to its pixel size if necessary so that all the final stimuli had the same ranged area.
2. *Density matching:* the density of a numeral array was defined as the ratio of the bounding circle surface and the numerocity. Therefore to control for density, the software was given a defined ratio which was translate to the diameter of the invisible circle in which the dots were positioned. The dots ware then bordered by a visible circle of 7° VA so to match all other stimuli.

Note – eventually all stimuli in all 3 condition were presented with a visible circle of 7°visual angle (so participant saw the same size of circle around all stimuli).
